# The pro-drug C13 activates AMPK by two distinct mechanisms

**DOI:** 10.1042/BCJ20240425

**Published:** 2024-09-12

**Authors:** Jordana B. Freemantle, Dinesh Shah, Dylan M. Lynch, Alessio Ciulli, Harinder S. Hundal, D. Grahame Hardie

**Affiliations:** 1 Division of Cell Signalling and Immunology, Sir James Black Centre, School of Life Sciences, University of Dundee, Dundee DD1 5EH, U.K.; 2 Centre for Targeted Protein Degradation, School of Life Sciences, University of Dundee, 1 James Lindsay Place, Dundee DD1 5JJ, U.K.

**Keywords:** AMPK, C13, formaldehyde, mitochondria, mitochondrial respiration

## Abstract

The AMP-activated protein kinase (AMPK) is a sensor of cellular energy status that is expressed in almost all eukaryotic cells. In the canonical activation mechanism, it is activated by increases in AMP:ATP and ADP:ATP ratios that signify declining cellular energy status. Once activated, AMPK phosphorylates numerous targets that promote catabolic pathways generating ATP, while inhibiting anabolic and other processes that consume ATP, thus acting to restore energy homeostasis. Pharmacological agents that activate AMPK have been useful in identifying downstream targets and have potential as drugs for treatment of metabolic disorders such as Type 2 diabetes and non-alcoholic fatty liver disease. One such agent is C13, a pro-drug with a phosphonate bis(isobutyryloxymethyl) ester moiety, with the isobutyryloxymethyl groups increasing membrane permeability. Following cellular uptake, C13 is cleaved to release C2, an AMP analogue and potent AMPK activator that is specific for complexes containing the α1 (but not the α2) catalytic subunit isoform. This has previously been assumed to be the sole mechanism by which C13 activates AMPK, with potential roles for the isobutyryloxymethyl groups being ignored. We now report that, following cleavage from C13, these protective groups are metabolized to formaldehyde, an agent that inhibits mitochondrial function and increases cellular AMP:ATP ratios, thus providing additional AMPK activation by the canonical mechanism.

## Introduction

AMP-activated protein kinase (AMPK) is the key component of a signalling pathway that is activated either by deprivation of cellular nutrients (especially glucose [1]) or of energy [2,3]. It acts in direct opposition to the mechanistic Target-of-Rapamycin Complex-1, a growth-promoting pathway that is activated under opposite circumstances, i.e. by the availability of nutrients and energy [4]. Once activated, AMPK phosphorylates over 100 downstream targets that, in general, switch on catabolic pathways or processes that generate ATP (including glycolysis, fatty acid oxidation and mitochondrial biogenesis), while switching off most biosynthetic pathways required for cell growth (including synthesis of fatty acids, sterols, nucleotides, ribosomal RNA and proteins) [2].

AMPK exists almost universally in eukaryotic cells as heterotrimeric complexes comprising catalytic α and regulatory β and γ subunits. In vertebrates each subunit is encoded by two or more genes, generating 7 subunit isoforms (α1, α2; β1, β2; γ1, γ2, γ3) that can form up to 12 heterotrimer combinations [5]. The canonical pathway for AMPK activation is triggered by changes in cellular adenine nucleotide levels. Stresses that deplete cellular energy status cause increases in ADP:ATP ratios, which are amplified by the adenylate kinase reaction (2ADP ↔ ATP + AMP) into even larger increases in AMP:ATP ratios [6]. The γ subunits of AMPK contain four tandem repeats of a sequence motif known (because two similar repeats occur in the enzyme cystathione β-synthase [7]) as CBS repeats. On AMPK these form three sites for binding of adenine nucleotides, with one potential site (CBS2) being unused. Displacement of ATP by AMP at the critical CBS3 site causes a large conformational change [8,9] that activates AMPK by three complementary mechanisms: (i) promoting phosphorylation at Thr172 within the activation loop of the α subunit kinase domain by the upstream kinase LKB1 [10,11]; (ii) protecting against Thr172 dephosphorylation by protein phosphatases [12]; (iii) allosteric activation of kinase already phosphorylated on Thr172 [13,14]. All three mechanisms can also be triggered by binding of ADP rather than AMP [15–17], although ADP may make only minor contributions to activation under physiological conditions [17].

Pharmacological activators have been useful in identifying targets and processes downstream of AMPK and have potential for development as drugs to treat metabolic disorders such as Type 2 diabetes or non-alcoholic fatty liver disease [18–21]. Currently known activators fall into three major classes according to their mechanism of action. Firstly, there are indirect activators that reduce ATP production either by inhibiting glycolysis (e.g. 2-deoxyglucose) or mitochondrial oxidative metabolism (e.g. the antidiabetic drug metformin). These drugs increase cellular AMP:ATP ratios and thus activate AMPK indirectly via the canonical mechanism [22]. Secondly, there are allosteric activators derived from high-throughput screens that bind the Allosteric Drug and Metabolite (ADaM) site, located in a cleft between the α and β subunits [23]. These include A-769662 [24], MK-8722 [19], PF-739 [20] and PXL-770, the latter being the first selective AMPK activator to have undergone a clinical trial in humans [21]. Some of these activators, including A-769662, are specific for AMPK complexes containing the β1 isoform and do not activate β2-containing complexes [25]. While most of these activators are synthetic molecules rather than natural products, long chain fatty acyl-CoA esters are naturally occurring ligands that allosterically activate β1-containing complexes by binding at the ADaM site [26]. Long chain acyl-CoA esters may represent feed-forward activators that promote ATP production via fatty acid oxidation when supply of glucose is limiting but fatty acids are available.

The third class of AMPK activators are pro-drugs that are converted into AMP analogues by cellular metabolism. The original example was 5-aminoimidazole-4-carboxamide ribonucleoside (AICA riboside or AICAR), which is taken up into cells by adenosine transporters and converted by adenosine kinase into the phosphorylated nucleotide AICA ribotide, often referred to as ZMP [27–29]. ZMP mimics all three effects of AMP on the AMPK system, although it is 50- to 100-fold less potent than AMP itself [29] and has known AMPK-independent effects, especially on other AMP-sensing enzymes such as glycogen phosphorylase [30] and fructose-1,6-bisphosphatase [31,32]. The use of AICA riboside as an AMPK activator should therefore now be avoided. Another pro-drug that is converted into an AMP analogue by cellular metabolism is C13 which, in place of the phosphate group of AMP, has a phosphonate group that is esterified at two positions with isobutyryloxymethyl groups [33]. C13 is taken up into cells and converted by cellular esterases into the AMP analogue C2. Remarkably, C2 is at least two orders of magnitude more potent as an allosteric activator of AMPK than AMP itself [33–35]. A crystal structure of the human α2β1γ1 complex revealed that two molecules of C2 bind in the CBS1 and CBS3 sites with their phosphonate groups making similar interactions to the phosphate groups of AMP, although their remaining 5-(5-hydroxyl-isoxazol-3-yl)-furan groups bind in a different orientation from the adenosine moieties of AMP [35]. C2 mimics at least two of the effects of AMP, i.e. allosteric activation and protection against Thr172 dephosphorylation by protein phosphatases, although both effects are specific for α1-containing AMPK complexes. Thus, complexes containing the α2 isoform display only very modest allosteric activation by C2, while C2 fails to protect against dephosphorylation at Thr172 [34].

Until now it has been assumed that all effects of C13 to activate AMPK in intact cells are mediated by C2, and possible roles for the isobutyryloxymethyl groups that are cleaved off by cellular esterases have not been considered. These two protecting groups are examples of acyloxyalkyl esters, whose enzymatic cleavage (most likely by carboxylesterases, paraoxonase or cholinesterases [36]) is known to give rise to transient hydroxymethyl intermediates, which then rapidly release formaldehyde [37]. Since C13 contains two isobutyryloxymethyl esters, two molecules of formaldehyde would potentially be released for each molecule of pro-drug metabolized. In this study, we confirm using a fluorescent probe based on rhodamine-6G [38] that formaldehyde is generated in cells treated with C13. This toxic product associates with mitochondria, and incubation of cells with either C13 or free formaldehyde leads to falling mitochondrial membrane potential (MMP), decreases in cellular oxygen uptake and increases in cellular AMP:ATP and ADP:ATP ratios. Our findings suggest that when designing pro-drugs containing esterified phosphonate moieties, careful consideration should be made of the blocking groups used to enhance membrane permeability.

## Results

### C13 treatment causes acetyl-CoA carboxylase phosphorylation in an AMPK-dependent manner

**Figure 1.**
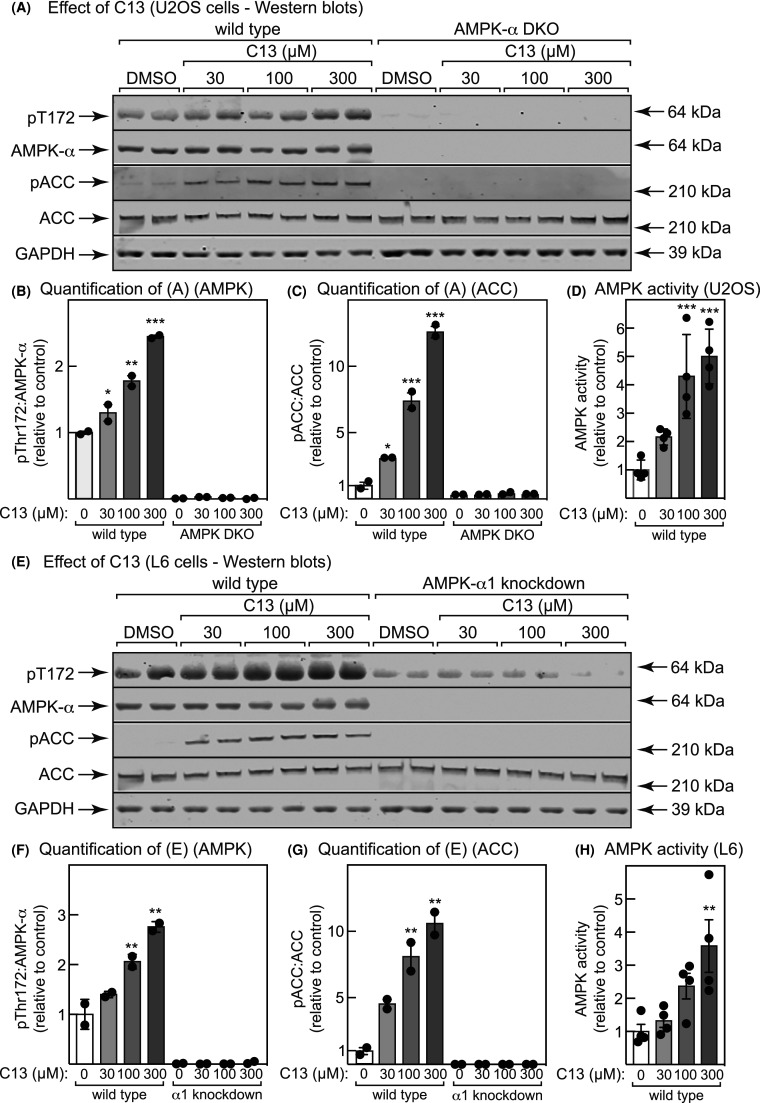
C13 activates AMPK in human and rat cells. (**A**) Human osteosarcoma (U2OS) cells, either wild type or double AMPK-α1/-α2 knockout made using CRISPR/Cas9 (DKO), were incubated with the indicated concentrations of vehicle (DMSO) or C13 for 1 h, cell lysates prepared and samples containing equal amounts of lysate protein analyzed by Western blotting with probing using antibodies against pThr172, total AMPK-α, pACC (pS80) or glyceraldehyde-3-phosphate dehydrogenase (GAPDH, loading control), or with streptavidin to detect total ACC. (**B**) quantification of (A) (pThr172/AMPK-α). (**C**) quantification of (**A**) (pACC/ACC). (**D**) AMPK activity measured in anti-α1/-α2 immunoprecipitates (wild type cells only). (**E**) Rat myoblast (L6) cells, either wild type or with AMPK-α1 knockdown made using shRNA, were incubated with the indicated concentrations of vehicle (DMSO) or C13 for 1 h, cell lysates prepared and samples containing equal amounts of lysate protein analyzed by Western blotting as in (**A**). (**F**) quantification of (**E**) (pThr172/AMPK-α). (**G**) quantification of (**E**) (pACC/ACC). (**H**) AMPK activity measured in anti-α1/-α2 immunoprecipitates (wild type cells only). For bar charts, results are mean ± SEM with individual points shown, with *n* = 2 for (**B**), (**C**), (**F**) and (**G**) and *n* = 4 for (**D**) and (**H**); mean values that are significantly different from DMSO controls by one-way ANOVA are indicated (*P < 0.05, **P < 0.01, ***P < 0.001).

Previous researchers have incubated intact cells with maximum concentrations of 100 µM C13 [34]. However, with either human osteosarcoma (U2OS) or rat myoblast (L6) cells, 100 µM was not saturating, because there was a further activation when the concentration was increased to 300 µM (Figure 1A/E). Thus, incubation of either cell type with 30, 100 or 300 µM C13 for 1 h yielded progressively increasing phosphorylation of Thr172 on AMPK (Figure 1B/F) and Ser79/80 on the AMPK target acetyl-CoA carboxylase (ACC) (Figure 1C/G), as well as increasing AMPK activity measured in anti-AMPK-α1/-α2 immunoprecipitates (Figure 1D/H). As expected, double knockout (DKO) of AMPK-α1 and -α2 using CRISPR:Cas9 in U2OS cells, or knockdown of AMPK-α1 using shRNA in L6 cells (α1 being the dominant isoform in those cells [39]) abolished AMPK-α expression. A trace of apparent Thr172 phosphorylation remained in the knockdown L6 cells, due either to residual α1 or to the α2 isoform. However, ACC phosphorylation (but not expression) was abolished in both the knockout U2OS and knockdown L6 cells, showing that the effects of C13 on this target were dependent on AMPK at all concentrations tested (Figure 1).

### Incubation of cells with C13 results in release of formaldehyde

**Figure 2.**
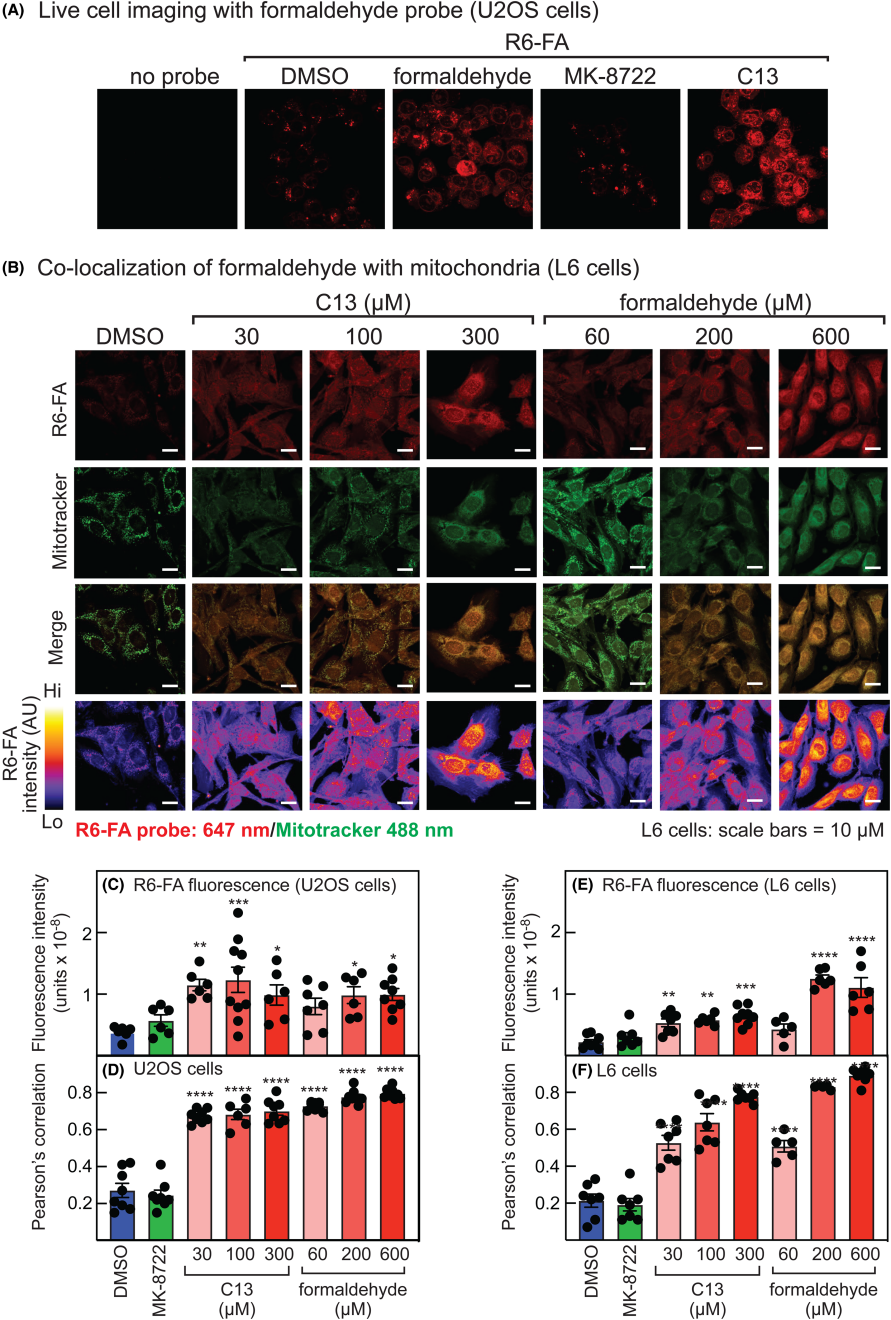
Formaldehyde is generated from C13 in U2OS cells, and associates with mitochondria. (**A**) U2OS cells were incubated with 5 µM R6-FA for 20 min and then with either vehicle (DMSO), formaldehyde (600 µM), MK-8722 (200 nM) or C13 (300 µM) for 1 h and analysed by fluorescence microscopy. (**B**) L6 myoblasts were incubated with 5 µM R6-FA and 200 nM Mitospy for 20 min prior to the addition of either vehicle (DMSO) or the indicated concentrations of C13 or formaldehyde for 1 h and analysed by fluorescence microscopy. The top three rows show fluorescence of the R6-FA (647 nm) or Mitospy (488 nM) probes or a merge of the two, while the bottom row shows false color images of the ratio of R6-FA fluorescence to that of Hoechst 3342 DNA-binding dye. (**C**) Fluorescence intensity of R6-FA in U2OS cells after the indicated treatments. (**D**) Pearson's correlation of fluorescence from R6-FA and the Mitospy mitochondrial marker in U2OS cells after the indicated treatments. (**E**) Fluorescence intensity of R6-FA in L6 myoblasts after the indicated treatments. (**F**) Pearson's correlation of fluorescence from R6-FA and Mitospy dye in L6 myoblasts after the indicated treatments. In (**C**) to (**F**), results are mean ± SEM (*n* = 5–10) with individual data points shown, and mean values that are significantly different from DMSO controls by one-way ANOVA are indicated (*P < 0.05, **P < 0.01, ***P < 0.001, ****P < 0.0001).

To investigate whether the cleavage of the C13 pro-drug to the AMP analogue C2 might result in the release of formaldehyde [37], we used an ultrafast illuminating fluorescent probe based on Rhodamine-6G (R6-FA), which becomes strongly fluorescent when it reacts with formaldehyde and can be used to detect formaldehyde in living cells [38]. When U2OS cells were incubated for 1 h with vehicle (0.1% dimethylsulfoxide, DMSO), live cell imaging revealed just a minor degree of aggregation of the fluorescent probe in the cell medium (Figure 2A). Similar results were obtained when cells were incubated with the ADaM site activator MK-8722 (200 nM), which would not be expected to liberate formaldehyde. However, when cells were incubated with 300 µM C13 or 600 µM free formaldehyde for 1 h, an obvious cytoplasmic fluorescence of the probe became evident, indicating the release or uptake of formaldehyde (Figure 2A) (we used twice the concentration of formaldehyde, because two molecules would potentially be released from each molecule of C13).

U2OS or L6 cells were next incubated with increasing concentrations of C13 or formaldehyde for 1 h, together with both the R6-FA probe and Mitospy dye, the latter to detect mitochondria. Very similar patterns of cytoplasmic fluorescence were obtained with both probes, and merged images suggested that formaldehyde was associating with mitochondria (Figure 2B shows results obtained with L6 cells). It was also clear that the amount of R6-FA fluorescence increased as the concentrations of C13 or formaldehyde were increased; false-color images to represent the intensity of R6-FA fluorescence in L6 cells (bottom row of panels in Figure 2B) showed that the signals increased as the C13 concentration was raised from 30 to 100 and 300 µM, and as the formaldehyde concentration was raised from 60 to 200 to 600 µM. Quantification of fluorescence intensity ratio (relative to the DNA-binding dye Hoechst 3342, Figure 2C,E) confirmed that similar progressive increases in R6-FA fluorescence were obtained with 30, 100 and 300 µM C13, and with 60, 200 and 600 µM formaldehyde in both U2OS and L6 cells, whereas the signal obtained upon treatment with another AMPK activator, MK-8722, was no different from that in a vehicle control. Analysis of Pearson's correlation between R6-FA and Mitospy fluorescence in U2OS or L6 cells (Figure 2D,F) revealed that treatment with C13 or free formaldehyde caused an increase from ∼0.2 in vehicle-treated and MK-8722-treated controls to 0.6–0.8. These results suggest that formaldehyde, whether released from metabolism of C13 or added to cells directly, associated with mitochondria.

### Treatment with C13 causes mitochondrial dysfunction

Formaldehyde can be liberated in the body either from drug metabolism or from ingestion of methanol, which is rapidly metabolized by alcohol dehydrogenases into formaldehyde, which is then converted to formate by aldehyde dehydrogenases [40]. At much higher concentrations than used here (10% formalin, which is 1.3 M formaldehyde in water containing 1% methanol), formaldehyde is used to fix cells for microscopy due to its ability to cross-link proteins and DNA [41]. At concentrations similar to those used here (up to 500 µM) exposure to formaldehyde for 24 h was found to be cytotoxic for SK-N-SH neuroblastoma or primary human foreskin fibroblasts, and reduced MMP and ATP levels in the former, while triggering mitochondrial fragmentation, reducing MMP and increasing expression of many mitochondrial genes in the latter [42].

**Figure 3.**
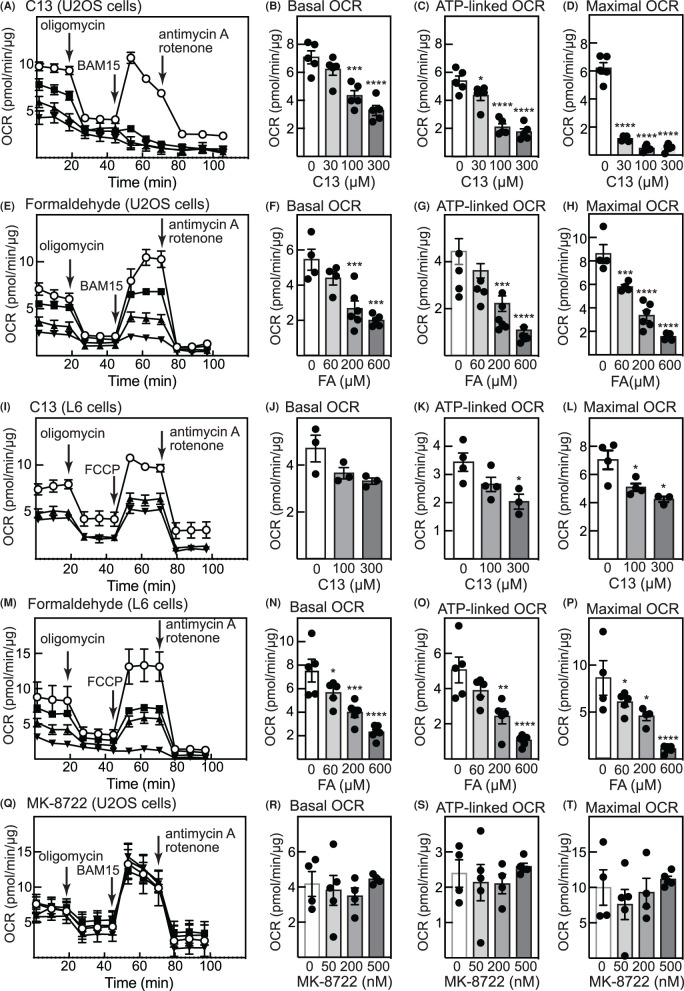
C13 and free formaldehyde both inhibit mitochondrial function in U2OS and L6 cells. (**A**) Effect of increasing concentrations of C13 added at time zero (open circles, DMSO control; filled squares, 30 µM; filled triangles, 100 µM; filled inverted triangles, 300 µM) on oxygen consumption rate (OCR) in U2OS cells. At the point shown by arrows, the ATP synthase inhibitor oligomycin (1 µM), the uncoupler BAM15 (U2OS cells, 1 µM) or FCCP (L6 cells, 1 µM), or the respiratory chain inhibitors antimycin A and rotenone (2 and 1 µM) were added as indicated and OCR recorded. (**B**) Effect of increasing concentrations of C13 on basal OCR (before addition of oligomycin) in U2OS cells. (**C**) Effect of increasing concentrations of C13 (data from (**A**)) on ATP-linked OCR (OCR_basal_ - OCR_oligomycin_) in U2OS cells. (**D**) Effect of increasing concentrations of C13 on maximal OCR (OCR_BAM15_ - OCR_antimycin/rotenone_) in U2OS cells. (**E**) As (**A**) but after treatment with formaldehyde (60, 200 and 600 µM). (**F**) to (**H**): as (**B**–**D**) but after treatment with formaldehyde (data from (**E**)). (**I**): as (**A**) but in L6 cells, in which FCCP was used in place of BAM15. (**J**) to (**L**): as (**B**) to (**D**) but with L6 cells (data from (**I**)). (**M**): as (**E**) but in L6 cells. (**N**) to (**P**): as (**F**) to (**H**) but in L6 cells (data from (**M**). (**Q**): as (**A**) but adding increasing concentrations of MK-8722 (30, 200, 500 nM) in place of C13 (U2OS cells). (**R**) to (**T**): as (**B**) to (**D**) but using MK-8722 (data from (**Q**)). For the bar charts results are mean SEM with individual data points shown (*n* = 3–6), and mean values that are significantly different from DMSO controls by one-way ANOVA are indicated (*P < 0.05, **P < 0.01, ***P < 0.001, ****P < 0.0001).

**Figure 4.**
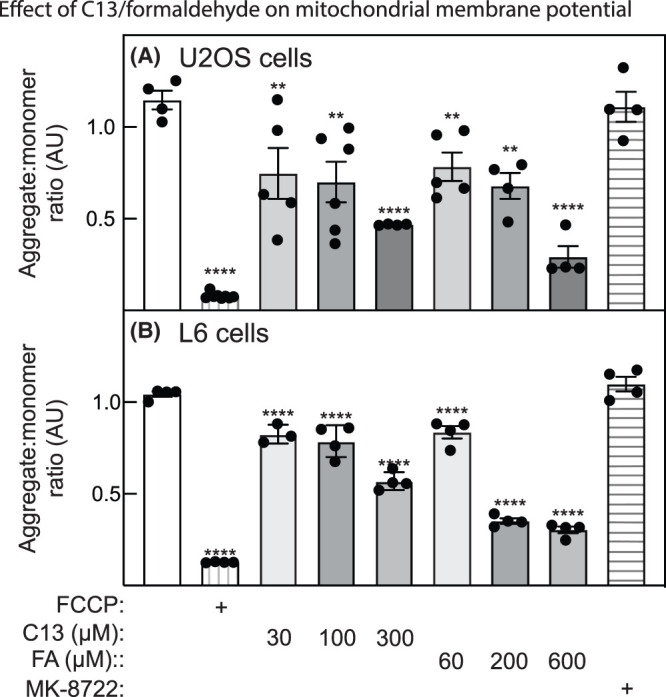
Effect of C13 or formaldehyde on mitochondrial membrane potential in (A) U2OS cells or (B) L6 cells. Cells were treated with the mitochondrial uncoupler FCCP (1 µM, positive control), with the ADaM site activator MK-8722 (200 nM, negative control), or with the indicated concentrations of C13 or free formaldehyde (FA). Results are mean ± SEM with individual data points shown (*n* = 3–8) and mean values that are significantly different from DMSO controls by one-way ANOVA are indicated (*P < 0.05, **P < 0.01, ***P < 0.001, ****P < 0.0001).

To investigate whether the generation of formaldehyde from C13 or direct treatment with formaldehyde would affect mitochondrial function in our cells, we performed extracellular flux analysis to measure basal, ATP-linked and maximal oxygen consumption rates (OCR) after treatment of U2OS or L6 cells with C13 or formaldehyde (Figure 3). Increasing concentrations of C13 or formaldehyde decreased basal OCR, ATP-linked OCR (measured as the difference between the basal OCR and the rate after addition of the F1-ATPase inhibitor, oligomycin) and maximal OCR (measured as the rate obtained immediately after addition of uncoupler, FCCP or BAM15). In contrast, the ADaM site activator MK-8722 did not significantly affect any of these measures. We also measured MMP using JC-10, a cationic, lipophilic dye that concentrates in healthy mitochondria as red fluorescent aggregates but returns to a monomeric green form in the cytosol if MMP collapses. As expected, treatment of U2OS or L6 cells with the uncoupler FCCP caused a collapse in MMP, while it was unaffected by the ADaM site AMPK activator MK-8722 (Figure 4). Treatment with increasing concentrations of C13 caused a progressive decrease in MMP, while treatment with equivalent concentrations of formaldehyde caused even larger decreases (Figure 4).

### Formaldehyde treatment of intact cells activates AMPK

**Figure 5.**
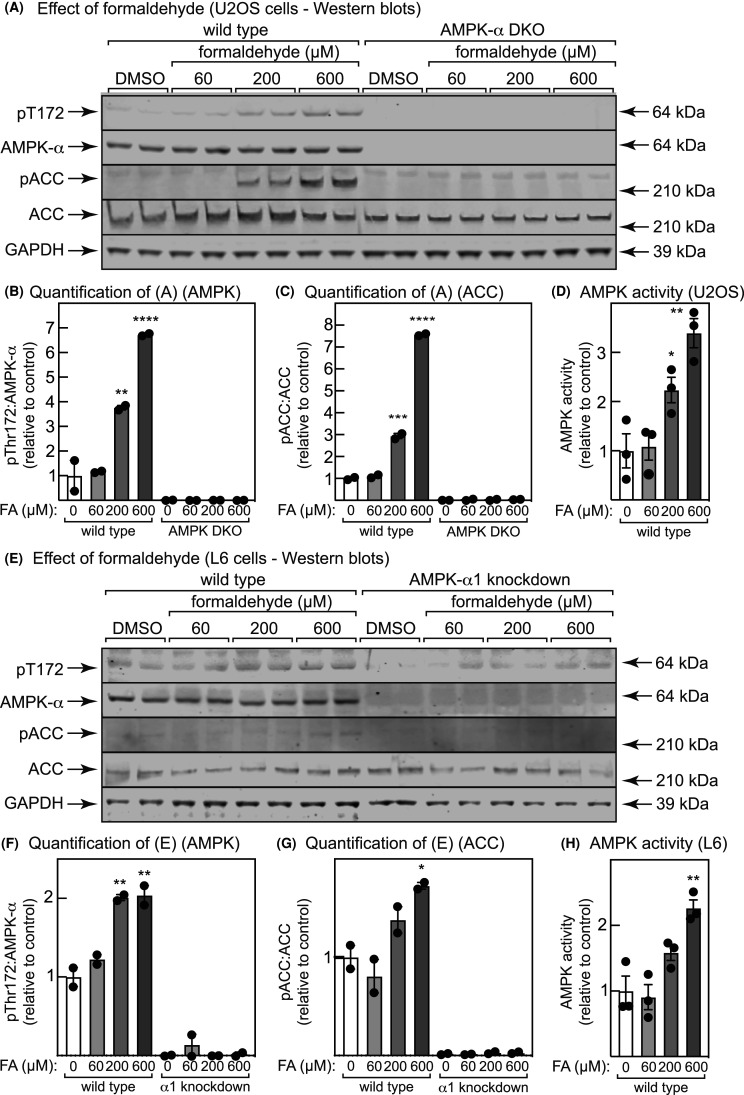
Effect of free formaldehyde on phosphorylation of AMPK and ACC and AMPK activity in U2OS and L6 cells. Wild type or AMPK-α1/-α2 double knockout (DKO) U2OS cells ((**A**) to (**D**)), or wild type or AMPK-α1 knockdown L6 cells ((**E**) to (**H**)) were incubated for 1 h with the indicated concentrations of formaldehyde and analyzed by Western blotting using the indicated antibodies or other probes. (**B**), (**C**), (**F**) and (**G**) show quantification of pT172 to total AMPK ratio and pACC (Ser79/80) to total ACC ratios, while (**D**) and (**H**) show AMPK activities measured in anti-AMPK-α immunoprecipitates. Results in bar charts are mean ± SEM with individual data points shown (*n* = 2 for Western blots and *n* = 3 for kinase assays), and mean values that are significantly different from DMSO controls by one-way ANOVA are indicated (*P < 0.05, **P < 0.01, ****P < 0.0001).

To test whether formaldehyde activates AMPK in the absence of C13, we treated WT or AMPK-α DKO U2OS cells with increasing concentrations of formaldehyde for 1 h. In WT cells this caused progressively increased phosphorylation of Thr172 on AMPK-α and of Ser80 on ACC, with both being absent in the DKO cells (Figure 5A–C). It also caused increased AMPK activity in the WT cells measured in anti-AMPK-α1/-α2 immunoprecipitates (Figure 5D). Very similar results were obtained in WT and AMPK-α1 knockdown L6 cells (Figure 5E–H).

### Treatment of cells with C13 or formaldehyde increases cellular AMP:ATP and ADP:ATP ratios

**Figure 6.**
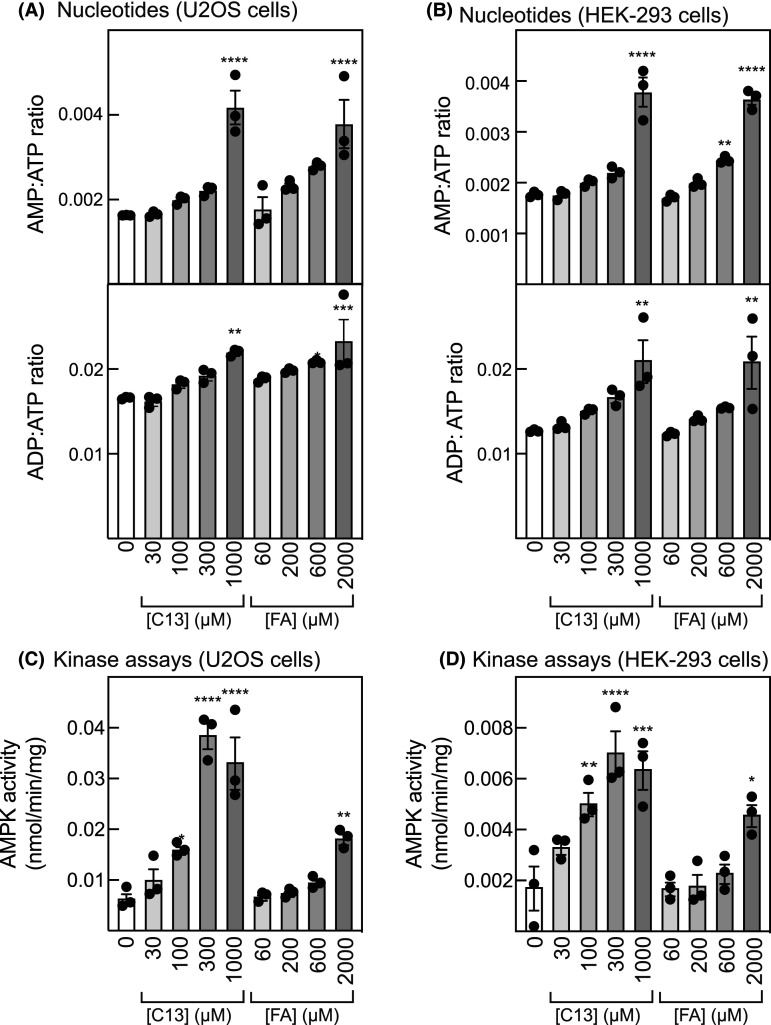
Effect of increasing concentrations of C13 and formaldehyde on nucleotide ratios and AMPK activities in U2OS or HEK-293 cells. (**A**) AMP:ATP and ADP:ATP ratios in U2OS cells after treatment for 1 h with the indicated concentrations of C13 or formaldehyde (FA). (**B**): as (**A**) but in HEK-293 cells. (**C**) AMPK activity assessed by immunoprecipitate kinase assay in U2OS cells. (**D**): as (**B**) but in HEK-293 cells. Results are mean ± SEM with individual data points shown (*n* = 3) and mean values that are significantly different from DMSO controls by one-way ANOVA are indicated (*P < 0.05, **P < 0.01, ***P < 0.001, ****P < 0.0001).

Since both C13 and free formaldehyde inhibited mitochondrial function, we expected that they would disturb energy status and thus increase cellular AMP:ATP and ADP:ATP ratios. This was indeed the case in U2OS cells and in another human cell line, HEK-293 cells (Figure 6A,B). Increases appeared to occur at all concentrations of C13 above 30 µM and formaldehyde above 60 µM, although they were only statistically significant at higher concentrations (1 mM C13, and 600 µM and 2 mM formaldehyde). As would be expected if the adenylate kinase reaction was at equilibrium [6], the increases in AMP:ATP ratio were larger than those in ADP:ATP. There were also significant increases in AMPK activity measured in an anti-AMPK-α immunoprecipitates in both cell types, which in the case of C13 were maximal at 300 µM, whereas the activity after formaldehyde treatment increased markedly as the concentration was raised from 600 µM to 2 mM (Figure 6C,D).

### Both C13 and formaldehyde fail to activate AMP-insensitive mutants of AMPK

**Figure 7.**
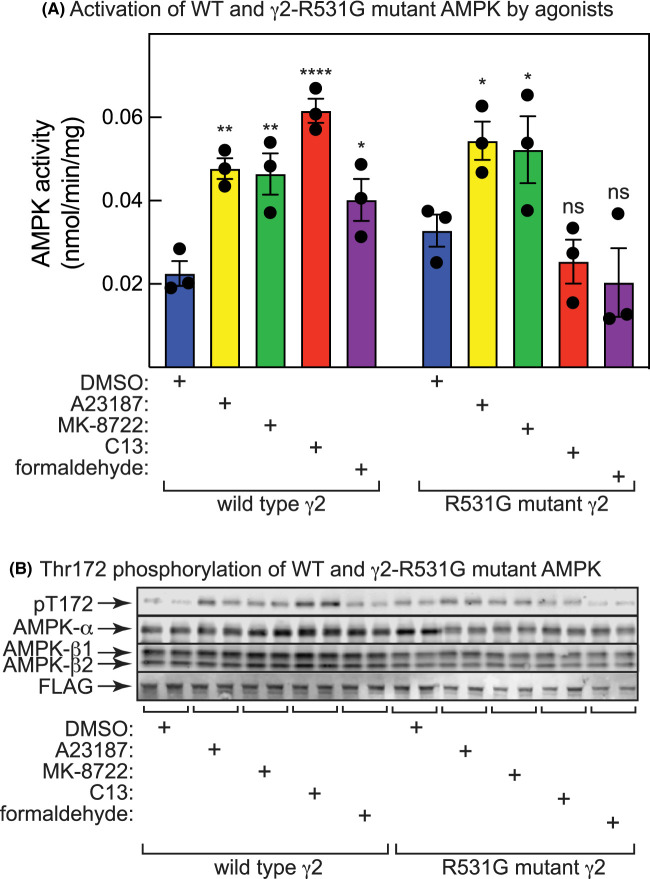
AMPK activation by both C13 and formaldehyde in HEK-293 cells are abolished by an R531G mutation in AMPK-γ2. Cells were incubated for 1 h with DMSO (control), A23187 (3 µM), MK-8722 (200 nM), C13 (300 µM) or formaldehyde (600 µM). (**A**) AMPK activity in anti-FLAG immunoprecipitates in HEK-293 cells expressing wild type γ2 or an R531G γ2 mutant. Results are mean ± SEM with individual data points shown (*n* = 3), and mean values that are significantly different from the DMSO controls for that genotype by two-way ANOVA are indicated (*P < 0.05, **P < 0.01, ****P < 0.0001, ns, not significant). (**B**) Phosphorylation of Thr172 on AMPK-α, and levels of AMPK-α, -β and -γ2 (FLAG-tagged) in HEK-293 cells expressing wild type γ2 or an R531G γ2 mutant.

To confirm that formaldehyde activated AMPK by increasing cellular AMP levels, we utilized HEK-293 cells and expressed by transient transfection a FLAG-tagged, wild type or AMP-insensitive mutant (R531G) of the AMPK-γ2 subunit. We have shown previously [22] in HEK-293 cells that the transfected γ2 subunit combines with endogenous AMPK-α and -β subunits, and that the recombinant WT or RG mutant complexes can be immunoprecipitated with anti-FLAG antibodies and their kinase activities measured. Since the RG mutant is insensitive to AMP, this approach can be used to test whether different agonists activate AMPK by canonical (AMP-dependent) or non-canonical (AMP-independent) mechanisms [22]. However, since the phosphonate group of C2 (the AMP analogue derived from C13 by cellular metabolism) binds to the same site as the phosphate group of AMP and interacts with Arg299 in γ1 [35] (equivalent to Arg531 in γ2), C2 also fails to activate complexes containing the R531G γ2 mutant [34]. Figure 7 shows that A23187 (a Ca^2+^ ionophore that activates AMPK by CaMKK2-dependent phosphorylation of AMPK-α Thr172), MK-8722 (which binds the ADaM site), C13 and formaldehyde all activated AMPK and enhanced phosphorylation of Thr172 with AMPK complexes expressing wild type γ2. However, while A23187 and MK-8722 still activated and increased Thr172 phosphorylation of complexes containing the AMP-insensitive R531G mutant of γ2, C13 and formaldehyde did not.

## Discussion

C13 is an isobutyryloxymethyl pro-drug containing an acyloxyalkyl class of ester-protecting group, which are commonly used protecting groups for phosphonates [26]. Upon cleavage of the isobutyryloxymethyl protecting groups by cellular esterases, such as carboxylesterases, paraoxonase, and cholinesterases, hydroxymethyl intermediates are transiently generated from which formaldehyde is rapidly released [37]. Since falls in ATP and/or MMP have been reported in SK-N-SH neuroblastoma cells and human primary foreskin cells treated with concentrations of formaldehyde similar to those used in this study [42,43], we examined whether any of the effects of C13 to activate AMPK could be explained by the generation in treated cells of formaldehyde, instead of or in addition to the known effects of the other product of cellular C13 metabolism, the AMP analogue C2. We provide in this study several lines of evidence that this is indeed the case. Firstly, treatment of human osteosarcoma (U2OS) or rat myoblast (L6 cells) with increasing concentrations of C13 caused enhanced cytoplasmic fluorescence of R6-FA, an ultrafast illuminating fluorescent probe for formaldehyde based on Rhodamine-6G. These results were similar to those seen in cells treated with free formaldehyde and were not seen in control cells treated with vehicle or the ADaM site activator of AMPK, MK-8722 (Figure 2). Secondly, the released formaldehyde appeared to be associated with mitochondria after treatment of cells with C13 or free formaldehyde, as indicated by increased Pearson's correlations from 0.2 to 0.6–0.8 between the fluorescence of R6-FA and Mitospy (Figure 2). Thirdly, C13 or free formaldehyde both appeared to inhibit the mitochondrial respiratory chain, as evidenced by decreases in basal, ATP-linked and maximal OCR measured by extracellular flux analysis (Figure 3), and by decreases in MMP measured in intact cells (Figure 4). Fourthly, treatment of U2OS or L6 cells for 1 h with free formaldehyde at molar concentrations twice those of C13 (since two molecules of formaldehyde would be generated from each molecule of C13) had similar effects on phosphorylation and activation of AMPK, and phosphorylation of its downstream target ACC, as did C13 (Figure 5). Fifthly, treatment of U2OS or HEK-293 cells with C13 or formaldehyde increased cellular AMP:ATP and ADP:ATP ratios and activated AMPK, especially at higher concentrations (Figure 6). Finally, C13 and formaldehyde activated recombinant AMPK complexes containing wild type γ2 expressed in HEK-293 cells but failed to activate complexes containing the AMP-insensitive R531G mutant of γ2. This contrasted with the effects of the Ca^2+^ ionophore A23187 or the ADaM site activator MK-8722, which activated both wild type and R531G complexes.

With respect to the last line of evidence, it should be noted that the other main product of cellular C13 metabolism, C2, binds to the AMPK-γ1 subunit at the crucial CBS3 site with its phosphonate group in the same location as the phosphate group of AMP, with both the phosphonate of C2 and the phosphate of AMP interacting with the side chain of Arg299 (equivalent to Arg531 in the γ2 isoform) [35]. Indeed, C2 has already been shown not to activate α1β1γ2 complexes containing the R531G mutation in cell-free assays [34]. Thus, the use of the R531G mutant in Figure 7 does not discriminate between whether activation by C13 is mediated by C2 or formaldehyde, or both. However, the fact that free formaldehyde fails to activate complexes containing the R531G mutation strongly suggests that the effects of C13 are at least partly mediated by formaldehyde, at least at higher concentrations — it is noticeable from Figure 6C,D that C13 appears to activate AMPK at lower equivalent concentrations (100 and 300 µM) than formaldehyde.

Our findings can explain some slightly puzzling findings from a previous study of the effects of C13 [34]. Although C2 had little or no effect on AMPK complexes containing the α2 isoform (when compared with the α1 isoform) in cell-free assays, it did activate α2-containing complexes in intact mouse hepatocytes, albeit at concentrations ∼10-fold higher than those required to activate α1-containing complexes. Moreover, C13 still inhibited synthesis of both saponifiable lipids (i.e. fatty acids) and non-saponifiable lipids (principally cholesterol) in AMPK-α1^−/−^ α2^+/+^ mouse hepatocytes despite the complete lack of the α1 isoform, although once again only at higher concentrations of C13. Taking our results together with those of Hunter et al. [34], at lower concentrations (<100 µM) C13 activates AMPK complexes by release of the AMP analogue C2, which binds to the AMPK-γ subunits in a different orientation than AMP [35], while at higher concentrations (>100 µM) it also activates AMPK by release of formaldehyde, which inhibits the mitochondrial respiratory chain and thus activates AMPK by the canonical (AMP-dependent) mechanism.

Despite this dual mode of AMPK activation, the effects of 300 µM C13 on phosphorylation of ACC at Ser79/Ser80 were completely dependent upon AMPK in both human (U2OS) and rat (L6 myoblast) cell lines, as shown by the lack of response in AMPK-α1^−/−^ α2^−/−^ DKO U2OS or AMPK-α1 knockdown L6 cells (Figure 1). However, in other cells and in studies of other downstream effects of AMPK activation, agents that deplete cellular ATP and thus activate AMPK by the canonical mechanism, including C13, might have off-target, AMPK-independent effects.

In summary, our results show that C13 can activate AMPK by two distinct mechanisms, i.e. production by cellular metabolism of: (i) C2, an AMP analogue and potent activator of AMPK, and: (ii) formaldehyde, which inhibits mitochondrial function and thus activates AMPK by increasing cellular AMP:ATP ratios. While the second, less specific mechanism can be partly alleviated in cell-based studies by using C13 at lower concentrations, for use as a human drug the production of the mitochondrial inhibitor formaldehyde would be undesirable, and other blocking groups to increase the cellular permeability of the phosphonate group should be considered.

## Materials and methods

### Chemicals and reagents

DMSO, dithiothreitol (DTT), (4-(2-hydroxyethyl)-1-piperazine ethane sulfonic acid) (HEPES), MgCl_2_, phenylmethylsulphonylfluoride (PMSF), soybean trypsin inhibitor (SBTI), tris(hydroxymethyl)methylamine (Tris), Triton X-100, Tween-20 and paraformaldehyde were from Merck KGaA (Hertfordshire, U.K.). HCl, NaCl, ethylene glycol-bis(2-aminoethyl ether)-N,N,N′,N′-tetraacetic acid (EGTA), Na ethylenediamine tetraacetate (EDTA), NaF, and Na pyrophosphate were from BDH (Lutterworth, U.K.). Orthophosphoric acid, glass slides and coverslips were from VWR (Lutterworth, U.K.). Adenosine monophosphate (AMP), adenosine triphosphate (ATP), and complete EDTA-free protease inhibitor cocktail were from Roche (Lewisham, U.K.). Glutathione-Sepharose beads and Optisafe HiSafe II liquid scintillation fluid were from PerkinElmer (Bucks, U.K.). Gibco Dulbecco's Modified Eagle's Medium (DMEM) GlutaMax™ high glucose, foetal bovine serum (FBS), trypsin-EDTA, Na pyruvate, ProLong™ Glass Antifade Mountant, NuPage® MOPS running buffer (20x, NuPage®), Tris-acetate running buffer, NuPage® pre-cast Novex 4–12% Bis-Tris gels, and NuPage® pre-cast Novex 3–8% Tris-acetate gels were from Life Technologies (Renfrewshire, U.K.). [γ-^33^P] ATP, disodium salt was from Hartmann Analytic (Braunschweig, Germany). 4′,6-Diamidino-2-phenylindole was from Vector Laboratories (Burlingame, CA, U.S.A.). *AMARA* peptide ([44], sequence AMARAASAAALARRR) was synthesised by Dr G. Bloomberg, University of Bristol.

### Cell culture and treatments

L6 myoblasts were cultured using α-minimal essential medium containing 2% (v/v) FBS. U2OS and HEK-293 cells were cultured using Dulbecco's Minimal Essential Medium (DMEM) with GlutaMax™ and high (25 mM) glucose containing 10% (v/v) FBS at 37°C and 5% CO_2_. Cells were washed using ice-cold PBS and lysed on ice at 80–90% confluence using lysis buffer (20 mM Tris–HCl pH 7.5, 50 mM NaCl, 2.5 mM Na pyrophosphate, 1 mM Na orthovanadate, 1 mM EDTA, 1 mM EGTA, 1 mM DTT, 0.1 mM benzamidine, 0.1 mM PMSF, 5 μg/ml SBTI, 1% (v/v) Triton X-100). U2OS cells with double AMPK-α (AMPK-α1/-α2) knockout and L6 cells with shRNA knockdown of AMPK-α1 were described previously [39,45].

### Immunoprecipitate kinase assays

These were carried out by lysing cells *in situ* on culture plates using ice-cold lysis medium that contained EDTA to quench protein kinase activities, as well as protein phosphatase inhibitors, proteinase inhibitors, and 1% (v/v) Triton X-100 [46]. AMPK was immunoprecipitated from lysate containing a known amount of total protein using a mixture of anti-α1 and -α2 antibodies coupled to protein G-Sepharose. AMPK was assayed in the resuspended immunoprecipitates by monitoring the transfer of radioactivity from [γ-^33^P]ATP to the *AMARA* peptide. The detailed methodology for the assay was described previously [47] except that we used [γ-^33^P]ATP rather than [γ-^32^P]ATP.

### SDS PAGE and immunoblotting

Samples were made to a final protein concentration of 1 mg/ml, mixed with 6x sample buffer, and heated at 95°C for 10 min using a QBT2 heating block. Bis-Tris 4–12% gels were run using NuPAGE™ MOPS buffer (50 mM 3-(N-morpholino)propanesulfonic acid (MOPS), 50 mM Tris base, 0.1% (w/v) SDS, 1 mM EDTA, pH 7.7) and used to analyze all proteins except ACC and pACC, when Tris-acetate 3–8% gels were run using Tris-acetate buffer (50 mM Tricine, 50 mM Tris base, 0.1% (w/v) SDS, pH 8.24). For cell lysates, total protein amount loaded was 30 μg. Gels were run using the Novex^TM^ Midi-Cell system for ≈90 min at 120 V, soaked for 5 min in 20% ethanol, and transferred using a BIO-RAD iBlot2™ onto 0.45 μM nitrocellulose membranes, which were then washed in Li-Cor Intercept™ (PBS) blocking buffer for 60 min. Membranes were then incubated with the following primary antibodies (all from Cell Signaling Technology) for 14–16 h at 4°C: ACC pSer79 (Cat #3661), AMPK-α1/-α2 (pThr172, Cat #2535L), pRaptor (pS792) (Cat #89146S), and Raptor (2280S). AMPK pan-α antibody (Cat #ab80039) was from Abcam. GAPDH (Cat #MAB374) antibody was from Sigma–Aldrich. After 3 × 10 min washes using TBS-T buffer, membranes were incubated with the following secondary antibodies for 60 min at room temp: IRDye® Li-COR Goat anti-rabbit 800 (P/N: 926-32211), IRDye® Li-COR Goat anti-mouse 700 (P/N: 926-68070), and Streptavidin DyLight™ 800 (Cat #21851) from Life Technologies. Imaging was performed using a Li-Cor Odyssey™ CLx and analysis and quantification using Image Studio Lite™ version 5.2.

### Extraction and mass spectrometry of cellular nucleotides

Nucleotide extractions and mass spectrometry were performed as described previously. Briefly, cells were lysed *in situ* on the culture plate in ice-cold 5% (w/v) perchloric acid, and perchloric acid was then extracted using tri-*n*-octylamine and 1,1,2-trichlorotriflouroethane [46]. The relative contents of AMP, ADP and ATP were determined in the neutralized extract by LC:MS (liquid chromatography:mass spectrometry) as described previously [1].

### R6-FA probe synthesis

FA probe: Chemicals were all commercially available, were purchased from Apollo Scientific, Sigma–Aldrich or Fluorochem and were used without further purification. Reactions were carried out under inert conditions, with the exclusive use of anhydrous solvents. Liquid chromatography-mass spectrometry (LC/MS) analyses were performed using either an Agilent Technologies 1200 series analytical high performance liquid chromatograph (HPLC) connected to an Agilent Technologies 6130 quadrupole LC/MS containing an Agilent diode array detector and a Waters XBridge C18 column (50 mm × 2.1 mm, 3.5 μm particle size), a Shimadzu HPLC/MS 2020 with photodiode array detector and Hypersil Gold column (1.9 μm 50 × 2.1 mm), or a Shimadzu HPLC LC-40B X3 with a photodiode array detector and Hypersil Gold column (1.9 μm 50 × 2.1 mm). Samples were eluted with a 3-min gradient of 5%–95% ACN:water containing either 0.1% formic acid (acidic method) or 0.1% ammonium hydroxide (basic method). NMR spectra were recorded on either a Bruker 500 MHz Ultrashield, or a Bruker 400 MHz Ascend spectrometer, and referenced to the residual solvent signal. Chemical shifts are reported in ppm and coupling constants (*J*) in Hz. Compounds were purified either by flash column chromatography using a Teledyne Isco Combiflash (Rf, Rf200i or NextGen 300+) with normal phase RediSep® Silica Gel disposable columns or reverse phase RediSep® Gold C18 columns. Methods were employed from [39,40].

Synthesis of 2-(2-aminoethyl)-3′,6′-bis(ethylamino)-2′,7′-dimethyl-spiro[isoindoline-3,9′-xanthene]-1-one (Supplementary Figure S1A): to a solution of Rhodamine 6G (958 mg, 2 mmol, 1 eq.) in EtOH (20 ml) at 50°C was added ethylenediamine (0.67 ml, 10 mmol, 5 eq.), and the resulting solution heated at reflux for 16 h, during which time the fluorescence disappeared. Following the elapsed time, the reaction was cooled to room temp, and the precipitate collected. The precipitate was washed with ice-cold EtOH (3 × 20 ml), followed by recrystallization from CH_3_CN. This gave the product as a fine white powder which was used without further purification (472 mg, 52%). The isolated compound was in good agreement with the literature [48]:

^1^H NMR (400 MHz, CDCl_3_) δ 7.96–7.88 (m, 1H), 7.50–7.39 (m, 2H), 7.09–7.02 (m, 1H), 6.34 (s, 2H), 6.23 (s, 2H), 3.50 (t, *J* = 5.2 Hz, 2H), 3.27–3.12 (m, 4H), 2.36 (t, *J* = 6.7 Hz, 2H), 1.90 (s, 6H), 1.32 (t, *J* = 7.1 Hz, 6H).

LCMS. t_R _= 1.61 min, over 3 min. *m/z:* C_28_H_33_N_4_O_2_ requires 457.26. Found: 457.24 [M + H]^+^

Synthesis of 2-(2-aminoethyl)-N3′,N6′-diethyl-2′,7′-dimethyl-spiro[isoindoline-1,9′-xanthene]-3′,6′-diamine (Supplementary Figure S1B): the lactam from the previous step (200 mg, 0.44 mmol, 1 eq.) was dissolved in anhydrous THF (5 ml), with a steady flow of N_2_ to continually blanket the flask. A solution of LiAlH_4_ (1 M in THF, 216 mg, 13 eq.) was added dropwise at 0°C. Following completion of the addition, the reaction turned gray, followed by light blue and then deep ultramarine. The reaction was stirred overnight and warmed to room temp. Following the elapsed time, the reaction was opened to air and quenched with dropwise addition of EtOH (10 ml), followed by H_2_O (100 ml). Vigorous evolution of gas was observed. The aqueous mixture was extracted with CH_2_Cl_2_ (3 × 20 ml), dried over Na_2_SO_4_, filtered, and concentrated *in vacuo*. Purification by reverse phase liquid chromatography (H_2_O + 0% CH_3_CN/H_2_O + 95% CH_3_CN, with 0.1% HCOOH as modifier) furnished the desired product as a pink solid following lyophilization (100 mg, 52%). The isolated compound was in good agreement with the literature [38]:

^1^H NMR (400 MHz, CDCl_3_) δ 7.30 (s, 1H), 7.24–7.16 (m, 1H), 6.92–6.82 (m, 1H), 6.42–6.31 (m, 4H), 4.19 (s, 2H), 3.33–3.16 (m, 5H), 2.70–2.62 (m, 1H), 2.51–2.36 (m, 2H), 1.99–1.93 (m, 6H), 1.32 (t, *J* = 7.1 Hz, 6H).

LC–MS: t_R _= 1.08 min, over 3 min. *m/z:* C_28_H_35_N_4_O requires 443.30. Found: 443.40 [M + H]^+^

### Seahorse mitochondrial stress test

Mitochondrial bioenergetics were measured in L6 myoblasts and U2OS cells using a Seahorse XF24 analyser. U2OS or L6 or cells, either wild type or mutant as indicated in Figures, were cultured on Seahorse culture plates and used to assess basal OCR, ATP-linked OCR, maximal OCR and non-mitochondrial OCR by injecting oligomycin (1 μM), FCCP (carbonyl cyanide p-trifluoromethoxyphenylhydrazone, 1 μM) or BAM15 (1 μM), rotenone (1 μM), and Antimycin A (2 μM) at the points indicated for ≈5 min prior to measurement of OCR. For some experiments, C13 was also injected, prior to the mitochondrial stress test compounds, to a final concentration indicated in Figure legends. OCR values were normalized to total cell protein.

### Live cell imaging

Cells were seeded into μ-Slide 8-well chamber slides (ibidia, U.K.) and washed with fresh PBS prior to incubation with 200 nM Mitospy Green FM (BioLegend, U.K.) and 5 μM R6-FA for 20 min prior to addition of compounds (C13 or formaldehyde) as indicated, and were maintained for 1 h until real-time imaging/visualization using a Zeiss 710 confocal microscope with a 60× oil-immersion objective at 37°C in a 5% CO_2_ chamber with excitation/emission set at 480 nm and 520 nm for Mitospy Green FM and 530 and 580 for R6-FA.

### Analysis of MMP

Cells were seeded and grown in dark-walled 96-well dishes until ≈90% confluent. They were carefully washed with PBS and treated with 1 µM FCCP (as a positive control to collapse mitochondrial potential) or with compounds (C13, MK-8722 or formaldehyde) as indicated, prior to further incubation with JC-10 (20 µM) at 37°C in a 5% CO_2_ atmosphere for 30 min. Fluorescence intensities were measured using excitation/emission at 490/525 nm (monomeric) and 540/595 nm (J-aggregates) of JC-10 using a CLARIOstar plate reader, prior to calculation of aggregate:monomer ratios.

### Statistical analysis

All statistical analysis was performed using GraphPad Prism® Version 10 for MacOS. Unless stated otherwise, results are presented as mean ± standard error of the mean (SEM), and one- or two-way analysis of variance (ANOVA) with the Holm-Sidak method for multiple comparisons was used to test significance of differences.

## Data Availability

Other than pilot studies, all data generated in this study has been included in this publication.

## Open Access

Open access for this article was enabled by the participation of University of Dundee in an all-inclusive *Read & Publish* agreement with Portland Press and the Biochemical Society under a transformative agreement with JISC.

## Abbreviations

ACC: acetyl-CoA carboxylase
ADaM: Allosteric Drug and Metabolite
AMPK: AMP-activated protein kinase
ANOVA: analysis of variance
DKO: double knockout
DMSO: dimethylsulfoxide
DTT: dithiothreitol
EDTA: ethylenediamine tetraacetate
FBS: foetal bovine serum
HPLC: High Performance Liquid Chromatograph
MMP: mitochondrial membrane potential
OCR: oxygen consumption rates
PMSF: phenylmethylsulphonylfluoride
SBTI: soybean trypsin inhibitor
SEM: standard error of the mean
