# Supplementary material for: The pro-drug C13 activates AMPK by two distinct mechanisms
Source: Biochem J. 2024 Sep 12;481(18):1203–19. doi: 10.1042/BCJ20240425 (PMC11555695; doi:10.1042/BCJ20240425)
Supplement: Supplementary Material 1 [file BCJ-481-1203-s1.pdf]

Fig. 1A: uncropped blots

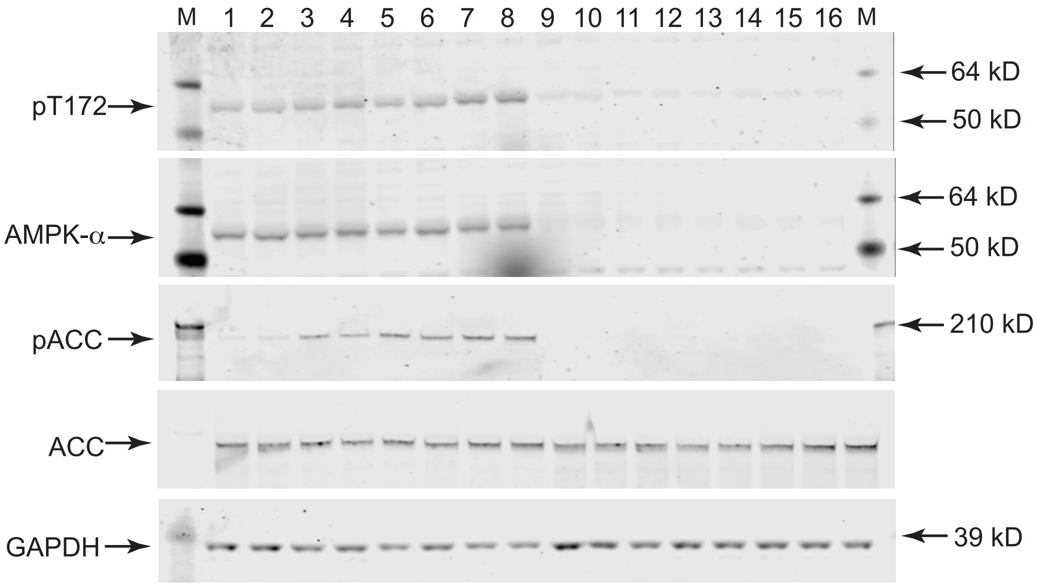

Fig. 1E: uncropped blots

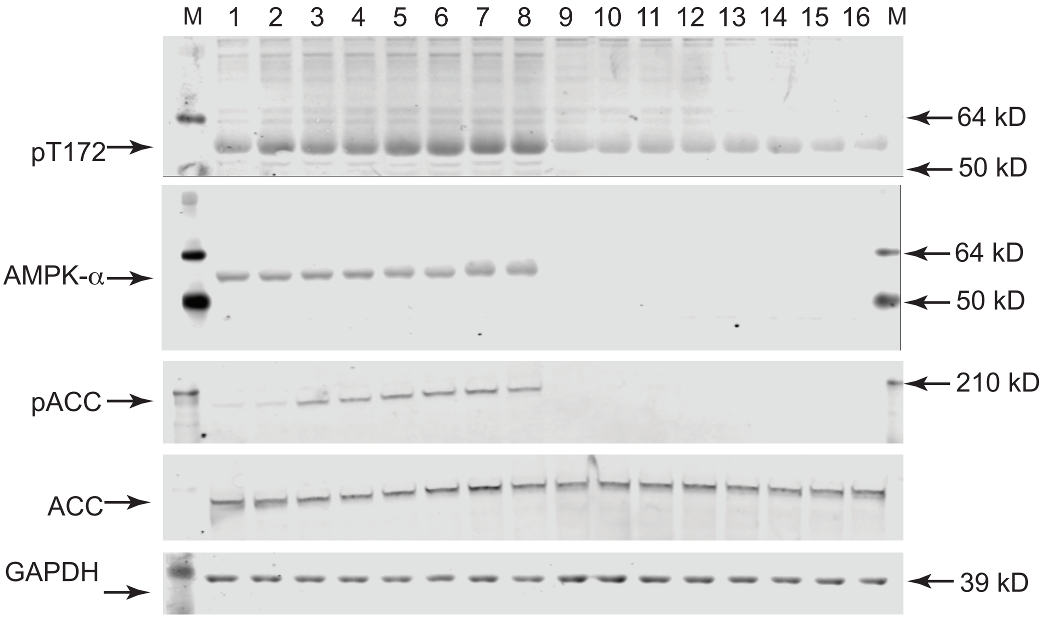

NOTES:

- to economise on sample, blots were often cut horizontally so that only the mass range relevant to the protein of interest was probed
- the molecular mass markers used (in lanes designated M) were SeeBlue Plus2 prestained protein standards (Cat. no. LC5925, ThermoFisher Scientific)
- molecular masses given for markers are those quoted by the manufacturer for the gel system used
- total ACC was detected using a streptavidin probe, which does not detect the markers
- total ACC was detected using a streptavidin probe, which does not detect the markers
- Invitrogen NuPAGE 3-8% Tris-acetate Midi-protein Gels were used to detect total and p-ACC; Invitrogen NuPAGE 4-12% Bis-Tris Midi-protein Gels were used to detect total and p-AMPK, and GAPDH; gels were run in Tris-acetate or MOPs running buffer, respectively, using the Invitrogen Novex Xcell4 Surelock Midi-Cell System
- electrophoretic transfer was performed using the Invitrogen iBlot2 Gel Transfer Device

Fig. 5A: uncropped blots

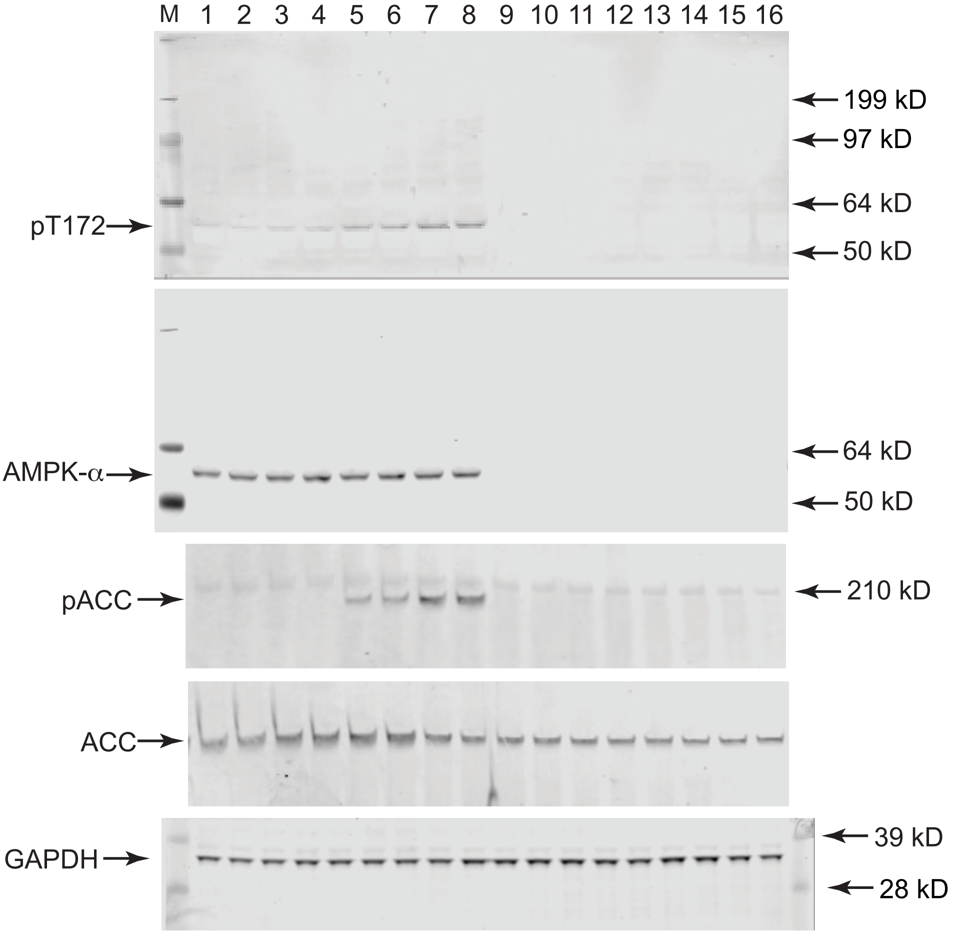

NOTES:

- to economise on sample, blots were often cut horizontally so that only the mass range relevant to the protein of interest was probed
- the molecular mass markers used (in lanes designated M) were SeeBlue Plus2 prestained protein standards (Cat. no. LC5925, ThermoFisher Scientific)
- molecular masses given for markers are those quoted by the manufacturer for the gel system used
- total ACC was detected using a streptavidin probe, which does not detect the markers
- Invitrogen NuPAGE 3-8% Tris-acetate Midi-protein Gels were used to detect total and p-ACC
- Invitrogen NuPAGE 4-12% Bis-Tris Midi-protein Gels were used to detect total and p-AMPK, and GAPDH

Fig. 5E: uncropped blots

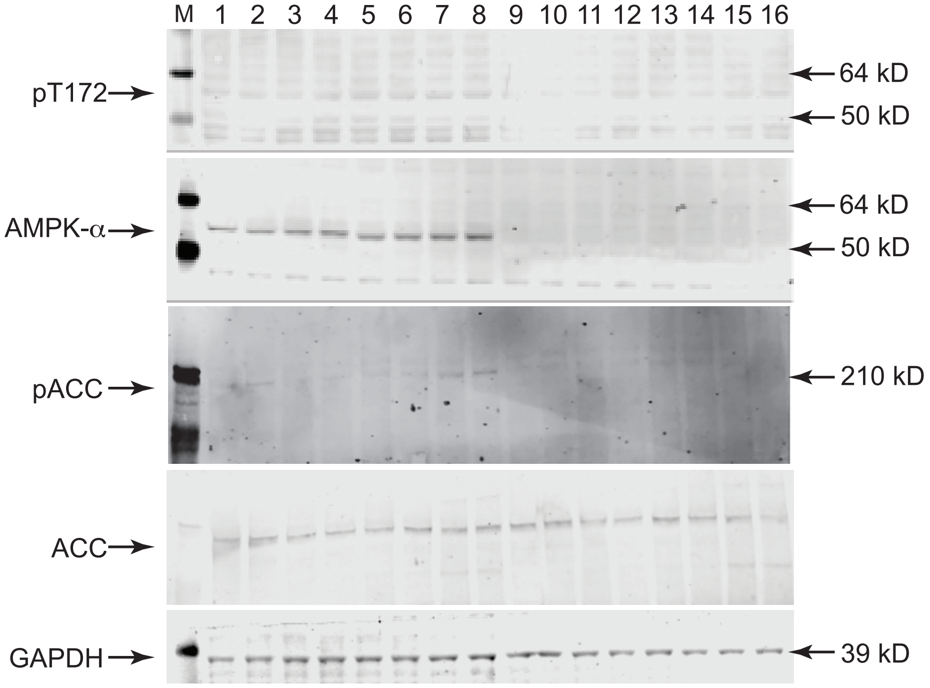

Fig. 7B: uncropped blots

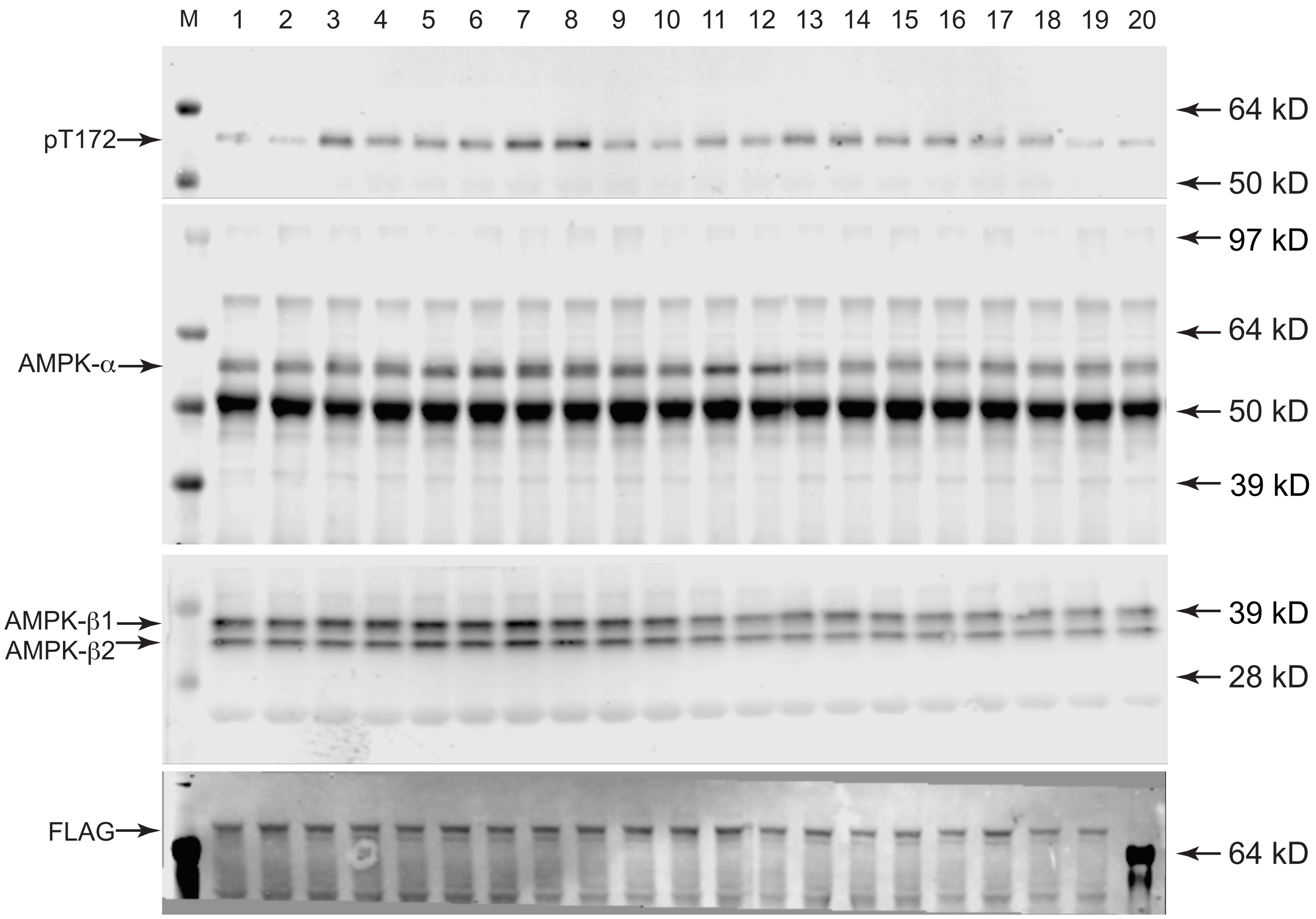

NOTES:

- to economise on sample, blots were often cut horizontally so that only the mass range relevant to the protein of interest was probed
- the molecular mass markers used (in lanes designated M) were SeeBlue Plus2 prestained protein standards (Cat. no. LC5925, ThermoFisher Scientific)
- molecular masses given for markers are those quoted by the manufacturer for the gel system used
- Invitrogen NuPAGE 4-12% Bis-Tris Midi-protein Gels were used to detect total and p-AMPK, AMPK-beta 1/2, and FLAG
